# Supplementary material for: Completing the ENCODE3 compendium yields accurate imputations across a variety of assays and human biosamples
Source: Genome Biol. 2020 Mar 30;21:82. doi: 10.1186/s13059-020-01978-5 (PMC7104481; doi:10.1186/s13059-020-01978-5)
Supplement: Supplementary file 1 — Additional file 1 Supplemental figures and tables. [file 13059_2020_1978_MOESM1_ESM.pdf]

# Additional File 1: ENCODE2018-Core results

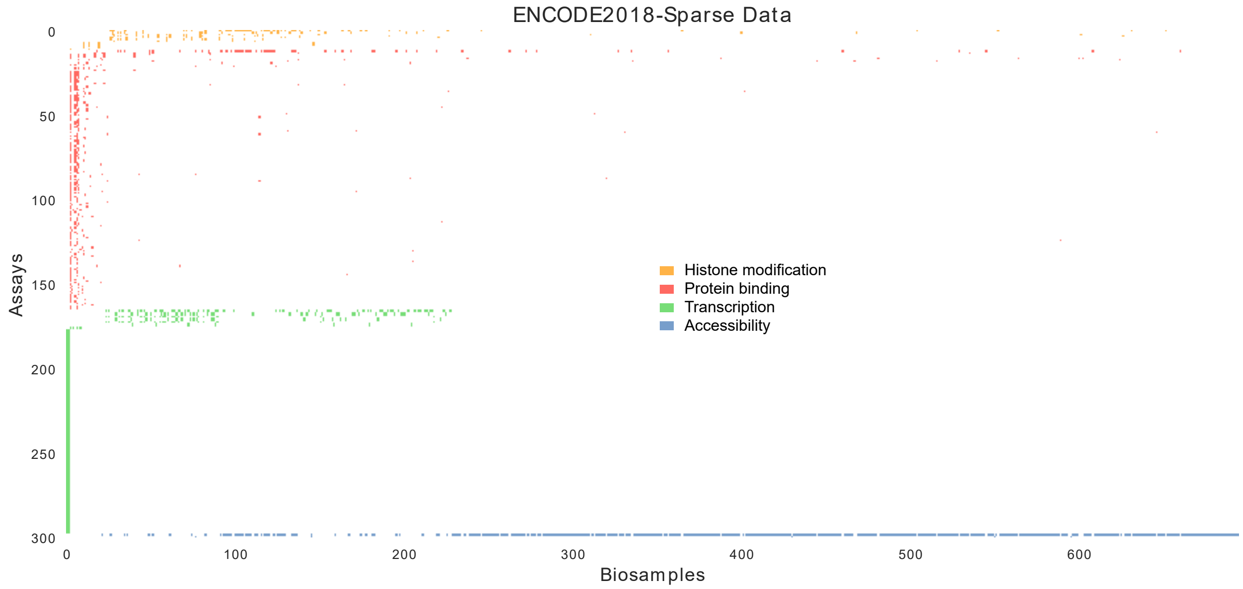


Figure S1: **The ENCODE2018-Sparse data matrix.** The ENCODE2018-Sparse data matrix includes all assays that were performed in fewer than 5 biosamples, and all biosamples that were characterized by fewer than 5 assays. Experiments that have been performed are displayed as colored rectangles, and experiments that have not been performed are displayed as white. The color corresponds to the general type of assay, with blue indicating chromatin accessibility, orange indicating histone modification, red indicating protein binding, and green indicating transcription. This figure displays all biosamples and the top 300 assays ranked number of biosamples that they were performed in.

| Biosample<br>Assay<br>Method | iPSC<br>CTCF | PC-3<br>CTCF | liver<br>EGR1 | liver<br>FOXA1 | liver<br>GABPA | liver<br>JUND | liver<br>MAX | liver<br>REST | liver<br>TAF1 |
|---|---|---|---|---|---|---|---|---|---|
| Yuanfang Guan | 0.655 | 0.564 | 0.433 | 0.341 | 0.355 | 0.535 | 0.473 | 0.386 | 0.320 |
| dxquang | **0.811** | 0.717 | 0.315 | 0.440 | 0.340 | 0.286 | 0.394 | 0.384 | 0.323 |
| autosome.ru | 0.709 | 0.458 | 0.364 | 0.323 | 0.360 | 0.441 | 0.434 | 0.353 | 0.261 |
| J-TEAM | 0.754 | 0.688 | 0.379 | **0.484** | 0.334 | 0.450 | 0.444 | 0.271 | 0.337 |
| Avocado | 0.665 | **0.724** | **0.542** | 0.401 | **0.431** | **0.630** | **0.570** | **0.513** | **0.425** |
| Similar Biosample | — | — | 0.410 | 0.437 | 0.257 | 0.581 | 0.500 | 0.457 | — |
| Same Biosample | 0.671 | 0.818 | 0.645 | 0.691 | 0.580 | 0.716 | 0.619 | 0.617 | 0.561 |
| Average Activity | 0.530 | 0.664 | 0.321 | 0.380 | 0.287 | 0.273 | 0.421 | 0.215 | 0.256 |

Table S1: **Comparison of methods on ENCODE-DREAM challenge test set.** The equal precision-recall (EPR) computed across nine epigenomic experiments in the ENCODE-DREAM challenge test set in chromosome 21. For each track, the score for the best-performing predictive model is in boldface.

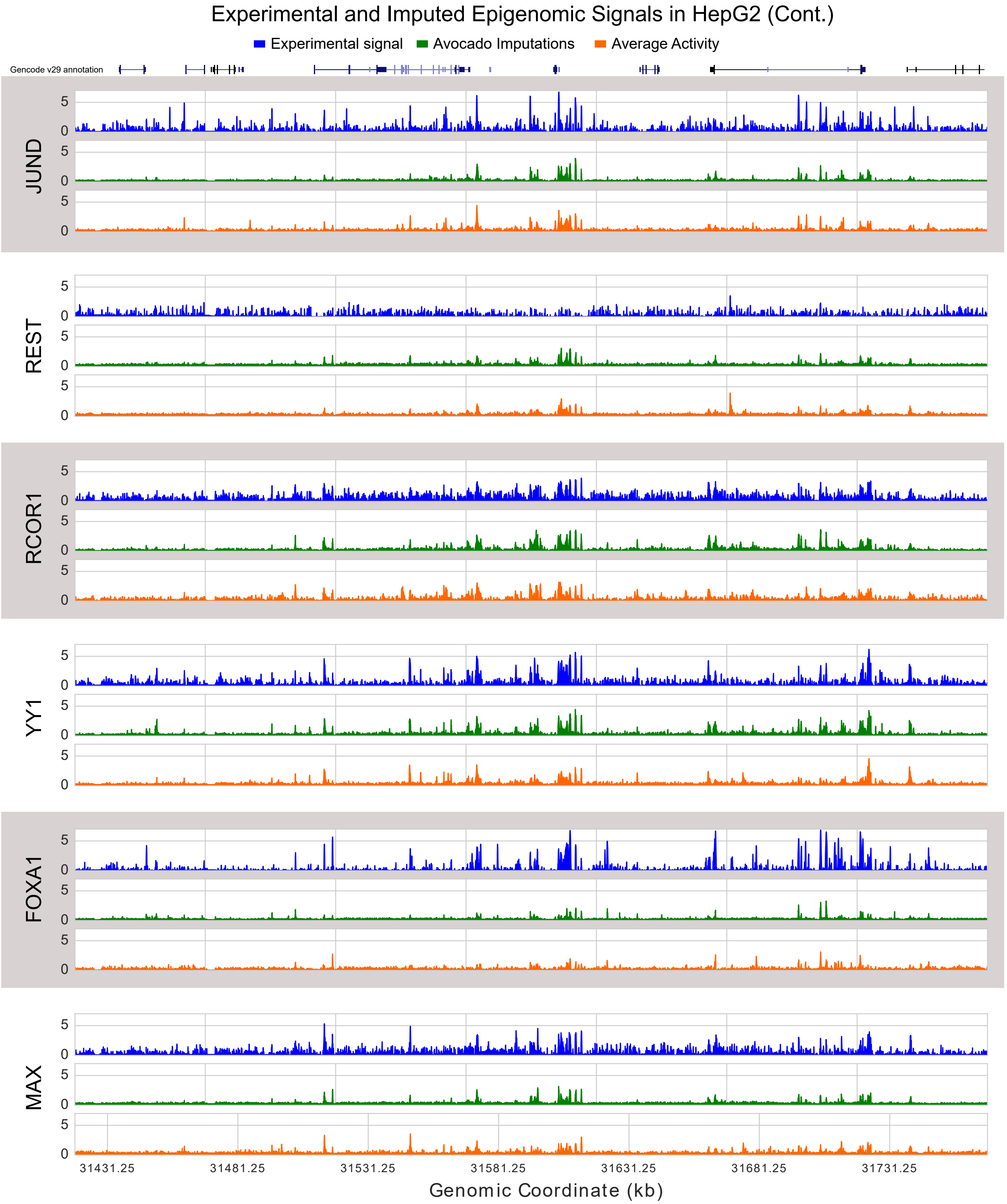


Figure S2: **Imputations of various transcription factors.** This figure extends Fig. 2a by showing the experimental signal (in blue), Avocado imputations (in green), and average activity baseline (in orange), for six additional transcription factors at the same locus.

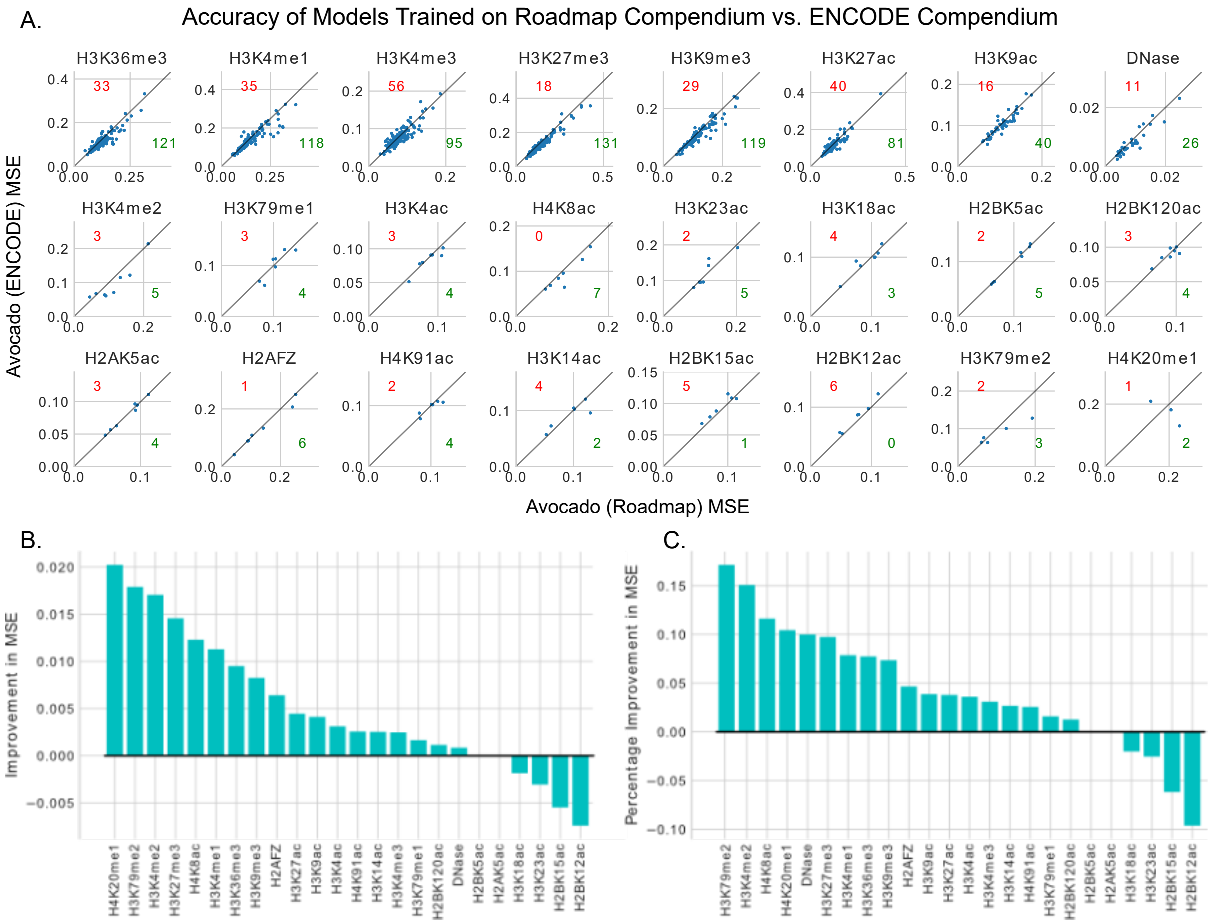


Figure S3: **Accuracies of models trained on either the Roadmap compendium or the ENCODE2018-Core data.** (A) Each panel depicts the error of models trained on either the ENCODE2018-Core dataset (Avocado (ENCODE)), or those tracks from the ENCODE2018-Core dataset that were provided by the Roadmap Epigenomics Consortium (Avocado (Roadmap)), when imputing the tracks contained in the latter. Each dot corresponds to MSE on a single track, and each panel corrsponds to all tracks from that assay. Dots below the diagonal line indicate that the model trained on the ENCODE2018-Core dataset outperformed the model trained on the Roadmap dataset, with the number in green specifying the number of such tracks, and dots above the line indicate the reverse, specified by the red number. (B) The improvement in performance when using a model trained on the full ENCODE2018-Core dataset versus one trained on only the Roadmap tracks. (C) Similar to (B), except the percentage improvement.

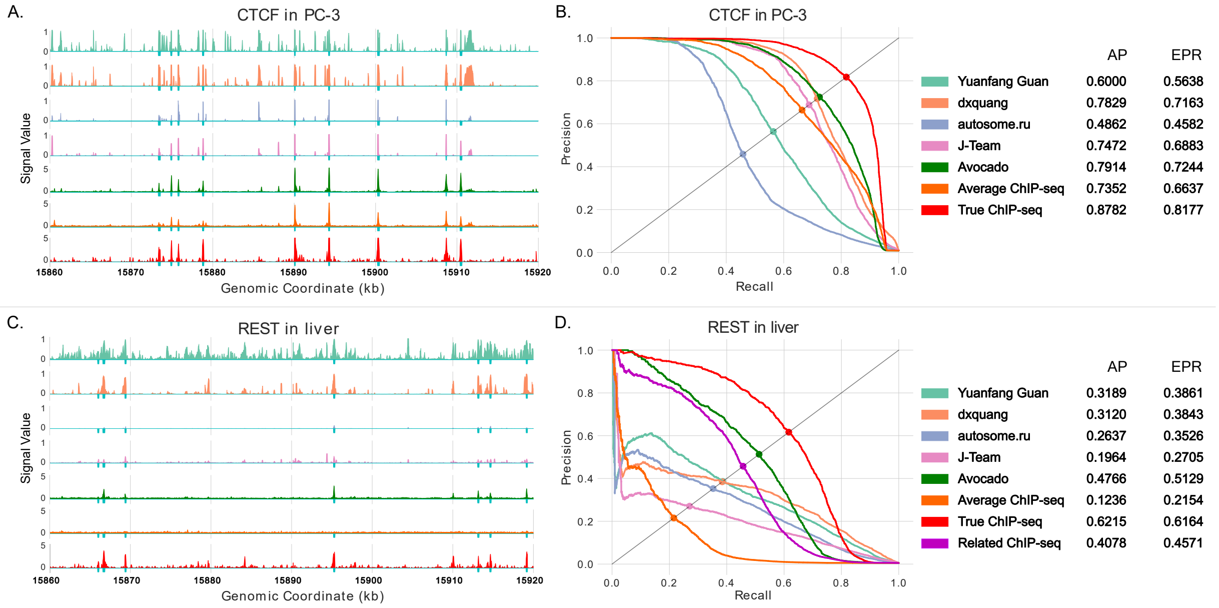


Figure S4: **Avocado imputes transcription factors correctly.** (A) Example predictions from a region of chromosome 21 for the top four ENCODE-DREAM participants, Avocado, and experimental ChIP-seq data measuring CTCF binding in PC-3. Cyan ticks at the bottom of the tracks indicate peak calls. (B) A precision-recall curve showing the performance of the four participants and Avocado in chromosome 21. As additional baselines, the experimental ChIP-seq signal (red) and the average signal across Avocado's training set (orange) were included in the comparison. For each approach, the average precision (AP) and the equal-precision-recall (EPR) are reported, and the position on the curve where the EPR lies is marked as a dot. (C) Similar to (A), except for REST binding in a liver biosample. (D) Similar to (B), except for REST binding in a liver biosample. The experimental signal from a different liver biosample is used as a further baseline (magenta).

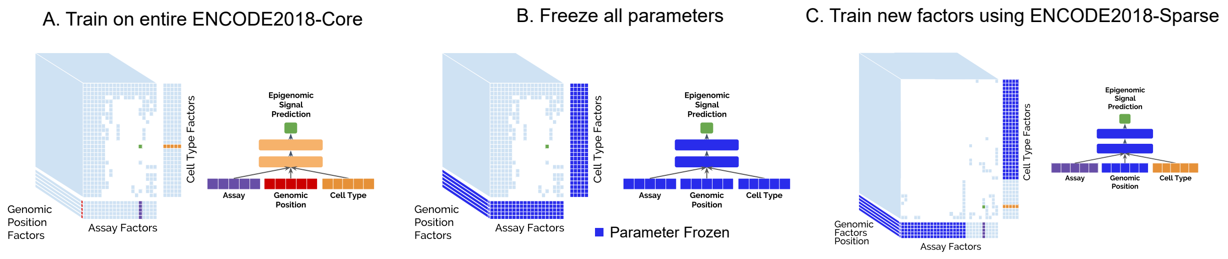


Figure S5: **Transfer learning methodology.** A schematic of the three step process to train Avocado on the ENCODE2018-Sparse dataset. (A) Train Avocado on the entire ENCODE2018-Core dataset as normal. (B) Freeze the weights of both the neural network and the factors. (C) Train only the factor values for new biosamples and assays that are being added to the model.

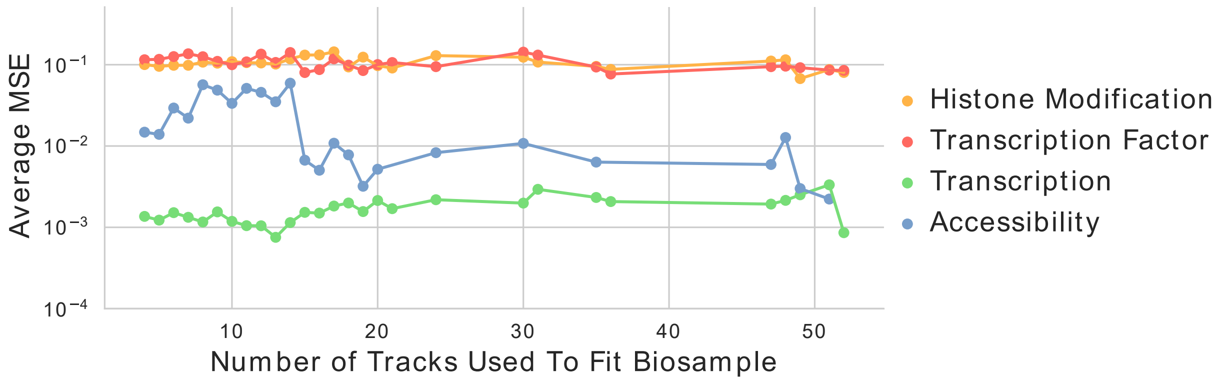


Figure S6: **Trends in imputation performance by number of assays per biosample.** The MSE of each of the 3,814 experiments in the ENCODE2018-Core data set averaged across both the number of assays used to fit the biosample factors of the experiment and the form of biological activity.

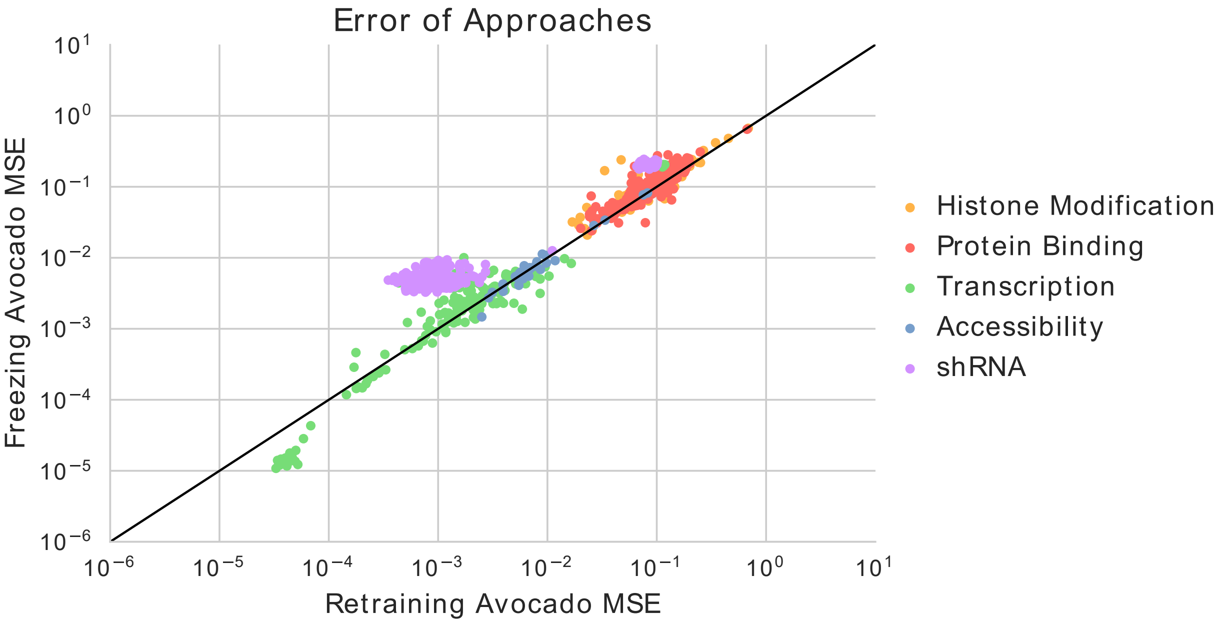


Figure S7: **Error of two methods for incorporating new experiments.** The MSE from each of 965 tracks of experimental data from the test set of ENCODE2018-Sparse from either retraining Avocado to include new experiments (x-axis) or freezing parameters from a pre-trained model and only training new biosample and assay factors (y-axis). The experiments are colored according to their type of biological activity.
