# Supplementary material for: Completing the ENCODE3 compendium yields accurate imputations across a variety of assays and human biosamples
Source: Genome Biol. 2020 Mar 30;21:82. doi: 10.1186/s13059-020-01978-5 (PMC7104481; doi:10.1186/s13059-020-01978-5)
Supplement: Supplementary file 2 — Additional file 2 Underperforming imputations. Follow-up analysis of those tracks whose imputations underperform the average activity baseline. [file 13059_2020_1978_MOESM2_ESM.pdf]

Additional File 2: Following up on experiments in which Avocado performs poorly

We investigated further the 13 experiments for which Avocado underperforms the average activity predictor. This set is enriched for measurements of transcription: 10 of the 13 experiments (77%) measure gene transcription, such as CAGE, RAMPAGE, microRNA-seq, polyA-depleted RNA-seq, and small RNA-seq. The remaining three assays for which Avocado does not outperform the average activity predictor according to mseGlobal are H3K9me2, EP300, and ATAC-seq. Further investigation on the ENCODE portal showed all H3K9me2 experiments had audit warnings and that only one of the experiments, in iPSC cells, had a fraction of reads in peaks (FRiP) score above the general quality control theshold of 1% used for ChIP-seq experiments [1]. While standards for ATAC-seq experiments have been released, the quality metrics associated with the experiments we used had not yet been released on the ENCODE portal, and so we were unable to verify their quality.

We then investigated those assays that Avocado underperformed the average activity baseline on other performance measures. First, we notice that Avocado imputed transcription poorly across all measures. On all measures except mse1imp, at least 9 of the underperforming assays related to measurements of transcription. Second, we notice that H3K9me2 and ATAC-seq are poor performers across all metrics as well. The consistent poor performance of these 11 assays may give a more pessimistic view of Avocado's performance in general.

We then evaluated assay performance across different performance measures. We noticed that Avocado only underperforms the average activity baseline on only five of the problematic transcription assays. This suggests that Avocado may have a higher precision than recall when it comes to predicting exon-specific activity. However, one weakness of mse1imp and mse1obs is that the percentage used to approximate peak coverage, 1%, may be appropriate for histone marks, but is not as specific to areas of transcription.

References

[1] Stephen G Landt, Georgi K Marinov, Anshul Kundaje, Pouya Kheradpour, et al. ChIP-seq guidelines and practices of the ENCODE and modENCODE consortia. *Genome Research*, 22(9):1813–1831, Sep 2012.
