# Supplementary material for: Completing the ENCODE3 compendium yields accurate imputations across a variety of assays and human biosamples
Source: Genome Biol. 2020 Mar 30;21:82. doi: 10.1186/s13059-020-01978-5 (PMC7104481; doi:10.1186/s13059-020-01978-5)
Supplement: Supplementary file 3 — Additional file 3 Further analyses of ENCODE challenge results. Follow-up analyses showing the performance of Avocado on the ENCODE TF Binding challenge when trained using different subsets of experiments to investigate the source of its strong performance. [file 13059_2020_1978_MOESM3_ESM.pdf]

Additional File 3: Further analyses of ENCODE challenge results

Comparing the performance of Avocado to the ENCODE Transcription Factor Binding Prediction Challenge participants is challenging for several reasons relating to differences in evaluation setting and model inputs. Accordingly, we performed several follow-up experiments to better understand the effect that these differences may have had on performance.

We began by characterizing the effect on predictive power produced by training models on the same loci that predictions were being made for. Described using the evaluation settings from Schreiber *et al.* [1], our original comparisons evaluated Avocado in the "cross-cell type" setting because the model had been trained and evaluated on the same chromosome, but we evaluated the challenge participants in the "hybrid" setting because their models had been trained and evaluated on different chromosomes. This does not mean that Avocado was evaluated on the training set, but rather that Avocado was evaluated on the ability to predict held-out tracks at the same loci that it was trained on, i.e., chromosome 21. When we evaluated all models using the cross-cell type setting by using chromosome 17, which was a part of the ENCODE challenge training set, we observed similar trends as in the original evaluation setting (Additional file 3: Table 1). This suggests that the evaluation setting was not a major confounder of performance.

Next, we investigated the extent to which Avocado leveraged the tracks of epigenomic data that were not available to the participants. This analysis involved training Avocado models in three settings. The first (denoted Avo0 in Additional file 3: Table 2) was to train Avocado on all tracks in the ENCODE2018-Core data set except for those in the challenge test set. This is in contrast to the evaluations presented in the main text, which are done on imputations made as a part of five-fold cross-validation. Because the model in this first setting was trained using more tracks than the models trained as a part of five-fold cross-validation, the resulting imputations should serve as an upper bound of performance for Avocado using the ENCODE2018-Core data set. The second setting (denoted Avo1) involved training Avocado using only DNase-seq and RNA-seq from the biosamples in the challenge, as well as the transcription factor binding tracks present in the training and validation sets of the challenge. In this setting, the model would have strictly less information than the challenge participants, who also had access to nucleotide sequence. The final evaluation setting was similar to the first setting, except that all tracks from biosamples in the challenge test set that were not DNase-seq and RNA-seq were also removed. This setting evaluates the ability of Avocado to leverage the ENCODE compendium to make imputations in biosamples while still using the same epigenomic input that the participants had.

Unfortunately, while it was simple to use the DNase-seq and RNA-seq experiments in our data sets for two biosamples (PC-3 and iPSC), there were several reasons why it was difficult to find corresponding experiments for the tracks denoted as "liver." The first difficulty is that the challenge test tracks actually come from two different liver biosamples: liver male adult 32 years (J099) and liver female child 4 years (J468), and Avocado treats these as distinct biosamples. The effect that including related data may have had was controlled for with the inclusion of the "similar biosample" row in Table 1. To further complicate matters, neither DNase-seq nor RNA-seq experiments had been performed in either of these liver biosamples. In the challenge, the RNA-seq track originates from a third, embryonic, biosample—liver female embryo 20 weeks and male embryo 22 weeks (J325)—and the DNase-seq track (`https://www.encodeproject.org/files/ENCFF530SFF/`) comes from a fourth biosample—right lobe of liver female adult 53 years (J288). To further complicate the comparison, the DNase experiment was revoked after the challenge and subsequently replaced with a higher quality experiment before we assembled the ENCODE2018-Core data set.

We addressed these difficulties in two ways. The first (denoted Avo2) was to simply remove J099, J468, and J325 from the model, and to use the RNA-seq and DNase-seq experiments from J288 (a biosample only present in the ENCODE2018-Sparse data set) as our new "liver" biosample. This approach ensured that there were matching DNase-seq and RNA-seq experiments from the same biosample, but had the drawback that neither experiment had been provided to the challenge participants nor were matched with the labels. The second approach (denoted Avo3) was to train two models, one to impute the tracks from J099 and one made to impute the tracks from J468. In each

Biosample<br>Assay<br>Method	iPSC<br>CTCF	PC-3<br>CTCF	liver<br>EGR1	liver<br>FOXA1	liver<br>GABPA	liver<br>JUND	liver<br>MAX	liver<br>REST	liver<br>TAF1
Yuanfang Guan	0.742	0.627	0.455	0.358	0.520	0.570	0.520	0.427	0.368
dxquang	**0.857**	0.800	0.358	**0.507**	0.470	0.283	0.407	0.396	0.355
autosome.ru	0.764	0.515	0.387	0.310	0.486	0.428	0.454	0.364	0.300
J-TEAM	0.812	0.767	0.421	0.480	0.465	0.441	0.426	0.266	0.346
Avocado	0.758	**0.856**	**0.571**	0.376	**0.542**	**0.692**	**0.676**	**0.585**	**0.542**
Similar Signal	0.731	0.685	0.427	0.417	0.293	0.557	0.571	0.494	0.217
Same Signal	0.768	0.924	0.706	0.740	0.696	0.763	0.734	0.718	0.647
Average Signal	0.634	0.796	0.435	0.335	0.384	0.364	0.437	0.386	0.363

Table 1: **Comparison of methods on chromosome 17 of the ENCODE-DREAM challenge test set.** The average precision computed across nine epigenomic experiments in the ENCODE-DREAM challenge test set in chromosome 17, which is one of the training set chromosomes. For each track, the score for the best-performing predictive model is in boldface.

Biosample<br>Assay<br>Method	iPSC<br>CTCF	PC-3<br>CTCF	liver<br>EGR1	liver<br>FOXA1	liver<br>GABPA	liver<br>JUND	liver<br>MAX	liver<br>REST	liver<br>TAF1
Yuanfang Guan	0.729	0.600	0.397	0.282	0.353	0.533	0.441	0.318	0.281
dxquang	**0.866**	0.783	0.274	0.399	0.347	0.260	0.330	0.311	0.264
autosome.ru	0.778	0.486	0.331	0.243	0.342	0.416	0.384	0.263	0.221
J-TEAM	0.812	0.747	0.363	**0.462**	0.344	0.415	0.377	0.196	0.272
Avocado	0.723	0.791	0.530	0.354	**0.396**	**0.660**	**0.574**	0.477	0.384
Avo0	0.733	0.779	**0.582**	0.430	0.381	0.650	0.550	**0.534**	**0.397**
Avo1	0.735	0.640	0.010	0.192	0.199	0.145	0.179	0.078	0.124
Avo2	0.788	**0.797**	0.105	0.088	0.242	0.117	0.112	0.145	0.112
Avo3	0.783	0.764	0.115	0.019	0.200	0.100	0.110	0.108	0.139
Similar Signal	0.627	0.570	0.363	0.389	0.226	0.568	0.446	0.408	0.096
Same Signal	0.741	0.878	0.648	0.716	0.573	0.731	0.622	0.622	0.556
Average Signal	0.574	0.736	0.324	0.299	0.253	0.375	0.336	0.327	0.197

Table 2: **Comparison of alternate Avocado methods on ENCODE-DREAM challenge test set.** The average precision for four alternate Avocado models (Avo0-Avo3) computed across nine epigenomic experiments in the ENCODE-DREAM challenge test set in chromosome 21. The numbers from Table 1 are also shown for comparison. For each track, the score for the best-performing predictive model is in boldface.

case, we fit the biosample that we are making predictions for using the DNase-seq and RNA-seq tracks from J288, and we fit the other biosample using include all of its assays. This evaluation setting has the benefit of measuring the effect that simply including data from a related liver biosample during training would have on model performance.

We observed the expected results in the first two settings (Avo0 and Avo1, Additional file 3: Table 2). In the first setting, the model either outperformed or exhibited comparable performance to the original Avocado model on each of the challenge test set tracks. In the second setting, the model performed very poorly on all tracks from the liver biosample, potentially due to the issues indicated above, and also performed worse than the original Avocado model at predicting CTCF in PC-3. Interestingly, we observed similar performance at predicting CTCF in iPSC as the first setting, despite having far fewer tracks as input. These results suggest that Avocado does indeed leverage the diversity of signals in the ENCODE compendium to make accurate predictions.

The third setting, where only DNase-seq and RNA-seq were used to fit the test set biosamples, generally showed similar results to the second setting. Specifically, both Avo2 and Avo3 underperformed the challenge participants on each of the tracks that were from liver. The mismatches in the DNase-seq and RNA-seq experiments denoted as liver are likely one reason for this poor performance, but it was difficult for us to assess whether they were the sole reason. Another potential reason for the poor performance was that the original Avocado model required the transcription factor binding signal from the related liver biosamples for reasonable performance, even if the model wasn't memorizing this signal. However, because Avo3—a model exposed to the transcription factor binding signal from the related liver biosample—still performed poorly, it seemed unlikely that this was a major reason. Interestingly, Avo2 achieved the highest performance at predicting CTCF of any Avocado model. The improvement in performance over Avo1 at predicting CTCF suggests that Avo2 was leveraging the epigenomic signal in the ENCODE compendium to make good predictions and that it is not necessary to have many assays for each biosample that one would want to make predictions in.

References

[1] J. M. Schreiber, R. Singh, J. Bilmes, and W. S. Noble. A pitfall for machine learning methods aiming to predict across cell types. *bioRxiv*, 2019. https://www.biorxiv.org/content/10.1101/512434v1.
