# Supplementary material for: Completing the ENCODE3 compendium yields accurate imputations across a variety of assays and human biosamples
Source: Genome Biol. 2020 Mar 30;21:82. doi: 10.1186/s13059-020-01978-5 (PMC7104481; doi:10.1186/s13059-020-01978-5)
Supplement: Supplementary file 4 — Additional file 4 The review history. [file 13059_2020_1978_MOESM4_ESM.docx]

Review History

**First round of review**

**Reviewer 1**

**Comments to author:**

This paper applies the method from a sister paper that was submitted to Genome Biology. It is very confusing to me how the original method could be efficiently applied to the entire genome. From the other paper, it only demonstrated that the method is working on a small subset of the chromosome. The issue with lab and processing protocol bias holds for this paper as well.

Additionally, I do not agree that directly applying another method to run on a large dataset without deriving substantial biological insights warrants a separate publication. It is more appropriately to be integrated into the other manuscript.

**Reviewer 2**

**Comments to author:**

The authors describe the application of an imputation approach (Avocado, described in a companion paper) for predicting a large number of missing (i.e., not yet measured) tracks from the ENCODE compendium. To demonstrate the high accuracy of their method, the authors attempt a comparison against the top performers of the recent ENCODE-DREAM challenge.
While I consider the tracks imputed by the authors' method of great utility for many applications, I have important reservations with regard to validity of the benchmarks provided in this paper, as explained below.


Major comments:

1. The manuscript is currently lacking details about the cross validation procedure mentioned on page 4, line 17 (especially how data are partitioned). Please add details to the Methods section.

2. From the current description in section 2.2 (page 6, line 42), I did not find an explanation why four transcription factors were excluded from the analysis (cf. Table 1), although data was available in the same manner as for the challenge participants. This is especially puzzling, since predictions for three of these factors are presented later in Figure 3.

3. As the authors point out themselves, the comparison to the ENCODE-DREAM participants is imperfect (page 7, line 40). In addition to the issues already mentioned by the authors (larger set of epigenomic features in training, training on the same genomic regions also made predictions for, not using sequence information, not trained for classification setting), challenge participants have been restricted to DNase-seq and RNA-seq data as epigenomic features in the target cell type. Notably, for the top approaches from the challenge, the transfer between genome versions would have been easily possible, since neither relied on specific parameters for genomic bins/positions. This also underlines the inherently different goals of the ENCODE-DREAM challenge (making predictions from a minimal set of assays, cf. https://www.synapse.org/#!Synapse:syn6131484/wiki/402032) and the Avocado approach (from my understanding: provide as accurate and complete imputations as possible).
For all these reasons, the "benchmark" against the ENCODE-DREAM participants is, in my opinion, too imperfect to allow for any major conclusions.
Either this benchmark could be replaced by a comparison to baseline models on the level of imputed tracks (like in the companion paper but possibly with a focus on TF tracks), or the most severe differences between the two settings need to be resolved. For instance, the authors could
i) perform the evaluation on a training region of ENCODE-DREAM (instead of chr21 from the test set),
ii) exclude all assays except DNase-seq and RNA-seq for the test cell type, and
iii) ideally (read: not necessarily) only use ChIP-seq measurements of the target transcription factor that were also available for challenge participants.

4. The statistical test comparing Avocado against the baseline (page 9 bottom, page 11 top, Figure 4B) seems to be flawed, and I would strongly recommend not to report a p-value in this setting. Specifically, the numbers are largely dominated by DNase data (475 tracks vs. 106 for all other assays), while training has been performed using DNase data. Using a Friedman test (or similar) for individual assays, likely only DNase and H3K4me3 would result in any significant difference.

5. The current version of the manuscript misses the opportunity to investigate two questions which, in my opinion, are important to assess the applicability of Avocado to new cell types and assays, also in the long(er) run.
First, the authors could demonstrate how much (or how little) in performance is lost by training only a small part of the parameter while the rest is "frozen" compared with a complete re-training. This information would also be important to decide (at some stage) if a re-training of the model could be worthwhile.
Second, the authors could investigate how many assays are necessary to yield "optimal" imputation performance. Is only DNase enough? How many histone modifications should be considered? Does methylation data help? Under the assumption that Avocado could become the standard for ENCODE imputation for at least a few years, this information would also be useful for experimental design.


Minor comments:

6. For illustrating the capabilities of Avocado, Figure 2 should also include one of the more complicated cases, e.g., JunD and/or REST (for both data are present for HepG2 in ENCODE).

7. For the comparison to participants of the ENCODE-DREAM challenge (section 2.2, Table 1), I would consider the area under the precision-recall curve (auPRC) instead of its approximation by AP the more natural choice, as auPRC has also been used in the challenge.

8. The color scales in most of the figures need to be adapted such that different tracks/groups/... are distinguishable also for colorblind individuals.

9. From the current description, it remained unclear to me if sub-figures 2B and 2C are also limited to HepG2 cells. Please add this information to the caption of Figure 2.

10. On page 7, line 25, the authors explain the good performance for CTCF with similar binding across cell types. Another important factor might be the (comparably) large number of CTCF binding sites, which leads to a less imbalanced classification problem than for other TFs.

11. For Figure 3, the information is missing on which chromosomes the evaluation has been performed.

12. On page 14, the authors note that 27% of chromosome 21 of hg38 could not be mapped to hg19. it would be important to know if the opposite is also true.

13. In Supp. Fig. S3 A/C, it would be interesting to also see a separate track for the average signal.

14. Typos, grammar, etc.
- page 2, line 37: double "been"
- page 9, line 23: "Figure 3a" should be "Figure 3".
- page 9, line 43: "Avvocado" -> "Avocado"
- page 9, line 56: "Supplementary Figure 4b" -> "Figure 4b"

**Authors Response**

**Point-by-point responses to the reviewers’ comments:**

Reviewer #1

This paper applies the method from a sister paper that was submitted to Genome Biology. It is very confusing to me how the original method could be efficiently applied to the entire genome. From the other paper, it only demonstrated that the method is working on a small subset of the chromosome. The issue with lab and processing protocol bias holds for this paper as well.

*As we described in the other response, Avocado operates in a genome-wide manner in both publications, and we do not believe that the bias issue is a significant factor in the data that we are using.*

Additionally, I do not agree that directly applying another method to run on a large dataset without deriving substantial biological insights warrants a separate publication. It is more appropriately to be integrated into the other manuscript.

*We discussed this issue with the editor and came to the conclusion that the two manuscripts will be considered for joint publication in a single issue of Genome Biology. The second manuscript introduces not just a larger data set but an entirely new task (predicting transcription factor binding).*

Reviewer #2

The authors describe the application of an imputation approach (Avocado, described in a companion paper) for predicting a large number of missing (i.e., not yet measured) tracks from the ENCODE compendium. To demonstrate the high accuracy of their method, the authors attempt a comparison against the top performers of the recent ENCODE-DREAM challenge. While I consider the tracks imputed by the authors’ method of great utility for many applications, I have important reservations with regard to validity of the benchmarks provided in this paper, as explained below.

*We thank the reviewer again for their careful consideration of our manuscript. We have modified the paper to incorporate the suggestions.*

Major comments:

1. The manuscript is currently lacking details about the cross validation procedure mentioned on page 4, line 17 (especially how data are partitioned). Please add details to the Methods section.

*We have added the following description of our partitioning procedure:*

*We adopted a similar strategy to Durham et al. for partitioning these experiments into folds for cross-validation. Specifically, we partitioned entire genome-wide experiments into five folds such that a model would be trained on all genomic loci and then evaluated on its ability to predict entirely held-out experiments, because this is the most realistic evaluation setting. However, randomly assigning tracks to each fold may inadvertantly leave some folds without seeing some assays or some biosamples, meaning that the model would not learn anything for those embeddings and thus perform poorly on imputation. Unfortunately, even after only keeping experiments from assays and biosamples where five experiments had been performed, it is not always possible to partition a set of experiments into folds such that each assay and biosample are seen. Thus, Durham et al. adopted a simple optimization approach that randomly assigned experiments to partitions and evaluated each partition by the total number of biosamples and assays covered by each partition. We empirically found that this approach underperformed a simple greedy approach that uses a counter to sequentially assign folds to random experiments within biosamples, one biosample at a time, preserving the location in the cycle from one biosample to the next.*

2. From the current description in section 2.2 (page 6, line 42), I did not find an explanation why four transcription factors were excluded from the analysis (cf. Table 1), although data was available in the same manner as for the challenge participants. This is especially puzzling, since predictions for three of these factors are presented later in Figure 3.

*We did not include those four transcription factors in the evaluation shown in Table 1 because those assays were not in the ENCODE2018-Core data set, which is the data set that we primarily used for evaluation of Avocado. These tracks were excluded from ENCODE2018-Core because, while there were experimental tracks available for those transcription factors, those assays had not been performed in at least five biosamples. The reason that some of these factors are present in Figure 3 is because it is showing the performance of the model at predicting TFs that had been added later to a pre-trained model. We have amended the text to make these points clearer.*

*Four of the assays, E2F1, HNF4A, FOXA2 and NANOG, were excluded from the ENCODE2018- Core data set because they had been performed in fewer than five biosamples. Consequently, Avocado could not make predictions for these four assays. Thus, we used only nine tracks for this evaluation.*

3. As the authors point out themselves, the comparison to the ENCODE-DREAM participants is imperfect (page 7, line 40). In addition to the issues already mentioned by the authors (larger set of epigenomic features in training, training on the same genomic regions also made predictions for, not using sequence information, not trained for classification setting), challenge participants have been restricted to DNase-seq and RNA-seq data as epigenomic features in the target cell type. Notably, for the top approaches from the challenge, the transfer between genome versions would have been easily possible, since neither relied on specific parameters for genomic bins/positions. This also underlines the inherently different goals of the ENCODE-DREAM challenge (making predictions from a minimal set of assays, cf. https://www.synapse.org/#!Synapse:syn6131484/wiki/402032) and the Avocado approach (from my understanding: provide as accurate and complete imputations as possible). For all these reasons, the “benchmark" against the ENCODE-DREAM participants is, in my opinion, too imperfect to allow for any major conclusions. Either this benchmark could be replaced by a comparison to baseline models on the level of imputed tracks (like in the companion paper but possibly with a focus on TF tracks), or the most severe differences between the two settings need to be resolved. For instance, the authors could i) perform the evaluation on a training region of ENCODE-DREAM (instead of chr21 from the test set), ii) exclude all assays except DNase-seq and RNA-seq for the test cell type, and iii) ideally (read: not necessarily) only use ChIP-seq measurements of the target transcription factor that were also available for challenge participants.

*The reviewer brings up the important point that the ENCODE-DREAM challenge comparison is imperfect. Please note that the primary goal of the comparison is to serve as an independent measure of the quality of the imputations. If models trained using a minimal amount of information could easily outperform Avocado’s imputations, then this might suggest that the imputations were not of high quality or that Avocado was not effectively utilizing the ENCODE compendium. However, we agree that further analysis might help clarify to which of the differences have a substantial effect on model performance. We have therefore performed the additional requested experiments and summarized them in Supplementary Note 2. Addressing point (i), we found that evaluating these models on chr17 (a training region in the ENCODE-DREAM challenge) yielded similar trends as evaluating the models on chr21. Points (ii) and (iii) were more difficult to address due to severe mismatches in liver biosamples present in the challenge, which we describe further in the Supplementary Note. In setting (ii) we find that Avocado still yields strong performance in non-liver biosamples but much poorer performance on the liver biosamples.*

4. The statistical test comparing Avocado against the baseline (page 9 bottom, page 11 top, Figure 4B) seems to be flawed, and I would strongly recommend not to report a p-value in this setting. Specifically, the numbers are largely dominated by DNase data (475 tracks vs. 106 for all other assays), while training has been performed using DNase data. Using a Friedman test (or similar) for individual assays, likely only DNase and H3K4me3 would result in any significant difference.

*While it is true that DNase-seq experiments were used to fit the biosample factors and so these factors may capture differences in DNase-seq better than other forms of activity, the evaluations are performed on a held-out chromosome, and so we reasoned that the evaluations are still fair. However, we do agree with the reviewer that the evaluations in DNase-seq are likely to dominate the p-value calculation, and so have removed it.*

5. The current version of the manuscript misses the opportunity to investigate two questions which, in my opinion, are important to assess the applicability of Avocado to new cell types and assays, also in the long(er) run. First, the authors could demonstrate how much (or how little) in performance is lost by training only a small part of the parameter while the rest is “frozen" compared with a complete re-training. This information would also be important to decide (at some stage) if a re-training of the model could be worthwhile.

*We agree with the reviewer that this is an important point and have evaluated three approaches for incorporating additional experiments. For clarity, we have split Section 2.3 into three parts, and included the results from these new analyses in Section 2.3.3. Overall, we found that fine-tuning a pre-trained model yielded the best performance when applied to new biosamples or assays, and that freezing parameters can lead to very poor performance on types of activity that were not seen on the original data set.*

Second, the authors could investigate how many assays are necessary to yield “optimal" imputation performance. Is only DNase enough? How many histone modifications should be considered? Does methylation data help? Under the assumption that Avocado could become the standard for ENCODE imputation for at least a few years, this information would also be useful for experimental design.

*Although the reviewer brings up the important point that, in practice, one may wish to tailor the ordering of experiments to yield the best imputations, we found this question difficult to easily address. The biggest obstacle is that it is computationally infeasible to consider combinations of input assays to quantify their relative performance. To address this problem, we averaged the performance of each track in the ENCODE2018-Core data set by both the number of assays performed in the training set for the relevant biosample and by the form of biological activity. The results are summarized in Supplementary Figure S6 (Figure 1 here).*

*While these evaluations have thus far used only DNase-seq to fit new biosamples to the model, it is not necessarily the case that performing a single assay is sufficient to optimally fit new biosamples to a model. Unfortunately, it would be computationally expensive to identify the combination of assays that yielded optimal performance. To investigate whether there was a general trend that biosamples fit with more assays performed better than those fit with fewer assays, we partitioned the 3,814 experiments from the five-fold cross-validation on ENCODE2018-Core by the number of assays performed in the biosample of the experiment (Supplementary Figure S6). When we plotted the average error of each type of activity, we did not observe a noticable trend between the number of assays used to fit biosample factors and performance at imputing experiments. This evaluation is limited by not considering the composition of experiments used to fit each biosample or by considering experiments that had fewer than four assays performed in the respective biosample. However, these results do not suggest that simply performing more experiments will yield better performance overall, or that biosamples with many experiments performed in them will necessarily have better imputations than those that are more sparsely assayed.*

Figure 1: Trends in imputation performance by number of assays per biosample. The MSE of each of the 3,814 experiments in the ENCODE2018-Core data set averaged across both the number of assays used to fit the biosample factors of the experiment and the form of biological activity.

Minor comments:

6. For illustrating the capabilities of Avocado, Figure 2 should also include one of the more complicated cases, e.g., JunD and/or REST (for both data are present for HepG2 in ENCODE).

*As suggested, we have included Supplementary Figure S2 (Figure 2 here) that show imputations for JUND, REST, RCOR1, YY1, FOXA1, and MAX in HepG2 at the same locus as Figure 2.*

7. For the comparison to participants of the ENCODE-DREAM challenge (section 2.2, Table 1), I would consider the area under the precision-recall curve (auPRC) instead of its approximation by AP the more natural choice, as auPRC has also been used in the challenge.

*As we mention in our previous response, average precision is a standard way for measuring the area under a precision-recall curve. While the challenge organizers use the Davis & Goadrich method for calculating the area, which can be more accurate with a small number of examples, in practice there is generally only a small difference when the number of examples is very large such as this case.*

8. The color scales in most of the figures need to be adapted such that different tracks/groups/... are distinguishable also for colorblind individuals.

*This turns out to be quite difficult to do in a way that accommodates all types of color deficiency and still yields a large enough pallette. We consulted several resources for designing color blind-aware schemes (colorbrewer2.org, https://usabilla.com/blog/how-to-design-for-color-blindness, https:// davidmathlogic.com/colorblind). Furthermore, one of the authors is color vision deficient, so we have a first-person test case. We are happy to work with the journal to find an appropriate color scheme consistent with their guidelines. Furthermore, if the reviewer has particular concerns about any specific figures, please let us know.*

9. From the current description, it remained unclear to me if sub-figures 2B and 2C are also limited to HepG2 cells. Please add this information to the caption of Figure 2.

*We have amended the caption to specify that it is all experiments over all biosamples.*

10. On page 7, line 25, the authors explain the good performance for CTCF with similar binding across cell types. Another important factor might be the (comparably) large number of CTCF binding sites, which leads to a less imbalanced classification problem than for other TFs.

*We agree with the reviewer that this point is worth explicitly discussing, and so have amended our discussion to include it.*

*This high performance is due in part to the large number of CTCF binding sites, but is likely also because CTCF binding is similar across most biosamples.*

11. For Figure 3, the information is missing on which chromosomes the evaluation has been performed.

*The evaluation was performed in chromosome 21, similar to the other comparisons against ENCODEDREAM participants. We have added this information to the caption.*

12. On page 14, the authors note that 27% of chromosome 21 of hg38 could not be mapped to hg19. it would be important to know if the opposite is also true.

*It is unclear to us why this would be important. In our evaluation we only use positions that could be successfully mapped between the two chromosomes, which is a symmetric relationship.*

*13. In Supp. Fig. S3 A/C, it would be interesting to also see a separate track for the average signal.*

*As requested, we have added a track corresponding to the average signal. Notably, the average ChIPseq signal for REST in the region we display is mostly low. When we visualized several other regions we noted better correlation between the average ChIP-seq signal (orange) and the true ChIP-seq signal (red), suggesting that this consistently low signal may be an artifact of having selected a region to visualize without considering the average ChIP-seq signal*.

14. Typos, grammar, etc.

- page 2, line 37: double “been"

- page 9, line 23: “Figure 3a" should be “Figure 3".

- page 9, line 43: “Avvocado" → “Avocado"

- page 9, line 56: “Supplementary Figure 4b" → “Figure 4b"

*We thank the reviewer for finding these mistakes. We have corrected them.*

Figure 2: Imputations of various transcription factors in HepG2. This figure extends Figure 2a by showing the experimental signal (in blue), Avocado imputations (in green), and average activity baseline (in orange), for six additional transcription factors at the same locus.

**Second round of review**

**Reviewer 1**

As mentioned in the other review, Avocado suffers from several fundamental logical errors. I listed the major ones.  It is not necessary to reiterate here.

Furthermore, the authors claim substantial improvement compared to ENCODE-DREAM methods. In the ENCODE DREAM challenge, entire chromesomes are held out; that method is deliverable to e.g. new species. In the author's method, the values in other cell lines on the test chromosome is available. In this case, even directly assign the value without any algorithm would perform much better than the ENCODE-DREAM best performing algorithms. By looking at the percentage of 'improvement' the authors reported, I believe it is worse than simply assigning the mean of all training cell lines.

**Reviewer 2**

Again, I am happy to state that the authors carefully addressed all of my concerns, leaving only a few minor comments I would like to make.

1. Regarding my previous comment 8, I would suggest the following
- in Fig. 2 A, Fig 4 A (and Fig. 2 B), the legend could also indicate the ordering of tracks (bars), since this would be enough to associate labels to tracks (bars)
- use the "conservative 7-color palette adapted for color blindness" from https://davidmathlogic.com/colorblind for Fig. 3

2. On page 22 and 23 (Supp Note 2), there seem to have been left (internal) comments in red.

3. Possible type:
Page 6, last sentence of 2.2: leverging -> leveraging

**Authors Response**

**Point-by-point responses to the reviewers’ comments:**

Reviewer #1

As mentioned in the other review, Avocado suffers from several foundamental logical errors. I listed the major ones. It is not necessary to reiterate here. Furthermore, the authors claim substantial improvement compared to ENCODEDREAM methods. In the ENCODE DREAM challenge, entire chromesomes are held out; that method is deliverable to e.g. new species. In the author’s method, the values in other cell lines on the test chromosome is available. In this case, even directly assign the value without any algorithm would perform much better than the ENCODEDREAM best performing algorithms. By looking at the percentage of ’improvement’ the authors

*We explicitly include the described baseline in our comparisons as “Average activity” in Table 1. We find that our method—and those of most of the challenge participants—generally outperform the baseline.*

Reviewer #2

Again, I am happy to state that the authors carefully addressed all of my concerns, leaving only a few minor comments I would like to make.

*We thank the reviewer again for their helpful feedback.*

1. Regarding my previous comment 8, I would suggest the following - in Fig. 2 A, Fig 4 A (and Fig. 2 B), the legend could also indicate the ordering of tracks (bars), since this would be enough to associate labels to tracks (bars) - use the "conservative 7-color palette adapted for color blindness" from https://davidmathlogic.com/colorblind for Fig. 3

2. On page 22 and 23 (Supp Note 2), there seem to have been left (internal) comments in red.

*Thank you for pointing this out; we have removed the comments.*

*3. Possible type: Page 6, last sentence of 2.2: leverging -> leveraging*

*We have fixed this typo.*
